# 1*α*, 25-Dihydroxyvitamin D_3_ and the vitamin D receptor regulates ΔNp63*α* levels and keratinocyte proliferation

**DOI:** 10.1038/cddis.2015.148

**Published:** 2015-06-11

**Authors:** N T Hill, J Zhang, M K Leonard, M Lee, H N Shamma, M Kadakia

**Affiliations:** 1 Department of Biochemistry and Molecular Biology, Boonshoft School of Medicine, Wright State University, 3640 Colonel Glenn Highway, Dayton, OH, USA; 2 Lifespan Health Research Center, Department of Community Health, Boonshoft School of Medicine, Wright State University, 3640 Colonel Glenn Highway, Dayton, OH, USA; 3 Department of Pediatrics, Boonshoft School of Medicine, Wright State University, 3640 Colonel Glenn Highway, Dayton, OH, USA; 4 Department of Dermatology, Boonshoft School of Medicine, Wright State University, 3640 Colonel Glenn Highway, Dayton, OH, USA

## Abstract

1*α*, 25-dihydroxyvitamin D_3_ (VD_3_), a secosteriod that has been explored as an anti-cancer agent, was also shown to promote cell survival. Its receptor, the Vitamin D Receptor (VDR), is a direct target of the proto-oncogene ΔNp63*α*, which is overexpressed in non-melanoma skin cancers. The interconnection between VDR/VD_3_ signaling and ΔNp63*α*, led us to examine whether VDR/VD_3_ signaling promotes keratinocyte proliferation by regulating ΔNp63*α* levels. Our data demonstrate that VDR regulates ΔNp63*α* expression at both the transcript and protein level. Interestingly, although low doses of VD_3_ led to an increase in ΔNp63*α* protein levels and keratinocyte proliferation, high doses of VD_3_ failed to increase ΔNp63*α* protein levels and resulted in reduced proliferation. Increased expression of ΔNp63*α* by low dose VD_3_ was shown to be dependent on VDR and critical for the proliferative effects of VD_3_. VD_3_-mediated increases in ΔNp63*α* protein levels occur via activation of both p38 MAPK and Akt kinases. Finally, analysis of samples from patients with squamous cell carcinoma (SCC), basal cell carcinoma and precursors to invasive SCC demonstrated a significant correlation between p63 and VDR levels when compared with healthy normal skin control samples. Delineation of the mechanisms by which VD_3_ exerts its effect on ΔNp63*α* and cell proliferation is critical for determining the future of VD_3_ in cancer therapies.

## Introduction

The Vitamin D Receptor (VDR) is a member of the nuclear receptor family. In canonical VD_3_ signaling, VDR bound to 1*α*, 25-dihydroxyvitamin D_3_ (VD_3_), the active form of Vitamin D_3_ will heterodimerize with the retinoic X receptor, thereby modulating the expression of its target genes involved in cellular proliferation or apoptosis by binding to vitamin D response elements in their promoter region.^1, 2^ VDR is known to localize in the mitochondria via the permeability transition pore independent of its ligand.^3^ VD_3_ has been explored as an anticancer agent because of its role in apoptosis and inhibition of angiogenesis.^4, 5, 6, 7^ VD_3_ has also been shown to promote cell survival via activation of the Akt pathway.^8, 9^ p38 was also activated in response to VD_3_.^10, 11^ Both increased Akt and p38 activity has been shown to increase the expression of ΔNp63*α*.^12, 13^

The transcription factor p63, a member of the p53 family, consists of six main isoforms due to alternative promoter usage and 3' splicing.^14^ The P1 promoter drives the expression of the full-length N-terminal transactivation domain isoforms, TAp63, whereas the use of an internal P2 promoter yields the transcription of the ΔNp63 isoforms harboring a truncated N-terminal transactivation domain. Differential 3' splicing of p63 yields *α*, *β* and *γ* isoforms of both TAp63 and ΔNp63 proteins.^14^

p63-null mice demonstrated that p63 is essential for the formation and proliferation of the epidermis along with other stratified epithelia.^15, 16, 17^ The most abundant and physiologically relevant p63 isoform, ΔNp63*α*, is widely expressed in the basal layers of stratified epithelium where it primarily functions in maintaining epithelial integrity.^15, 18, 19, 20, 21, 22^

ΔNp63*α* is overexpressed in many human cancers including non-melanoma skin cancers (NMSCs) such as basal cell carcinomas (BCC) and squamous cell carcinomas (SCC).^18, 23, 24, 25, 26, 27, 28^ However, the loss of ΔNp63*α* leads to increased cell invasion.^29, 30^ Little is known about the mechanism underlying p63 regulation, particularly in the skin epithelium.

In this study, we examined whether VD_3_ and VDR promotes keratinocyte proliferation via the regulation of ΔNp63*α* expression. We demonstrate that VDR positively regulates the expression of ΔNp63*α*. Furthermore, VD_3_ has a dose-dependent effect on ΔNp63*α* protein level. A direct correlation was observed between VD_3_-mediated increase in ΔNp63*α* levels and keratinocyte proliferation, which is dependent on VDR. Inhibition of both Akt or p38 activation led to a reduction in VD_3_-mediated increase in ΔNp63*α* protein levels. We observed significantly higher levels of both p63 and VDR expression in NMSCs when compared with normal skin indicating a possible correlation between p63 and VDR in these cancers.

## Results

### VDR is essential for basal expression of ΔNp63*α*

**Figure 1:**
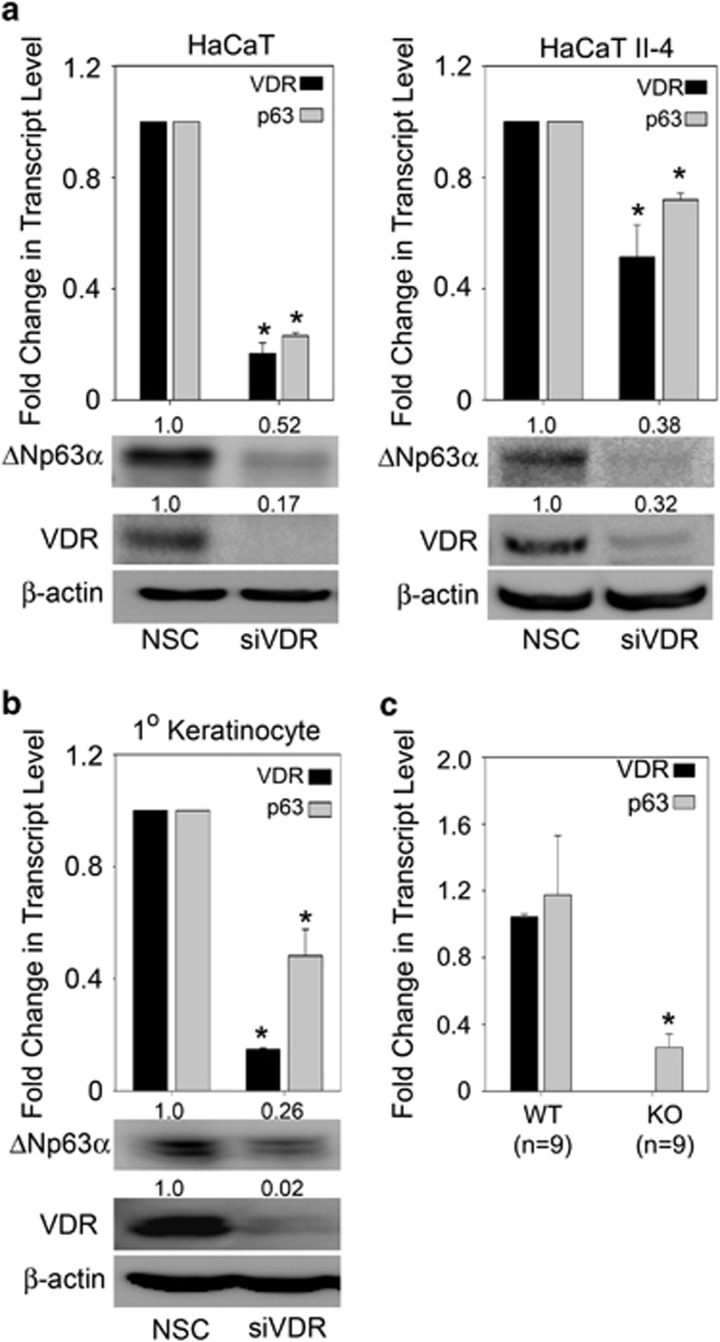
VDR is essential for basal expression of ΔNp63*α*. (**a**) HaCaT (left panel), HaCaT II-4 (right panel) and (**b**) neonatal human epidermal keratinocyte cells were transfected with non-silencing control (NSC) or siRNA against VDR. The change in mRNA levels and protein expression of p63 and VDR were measured by qRT-PCR (*P values≤0.05) and immunoblot analyses, respectively. (**c**) The change in transcript levels of p63 and VDR were measured by qRT-PCR in total RNA extracted from skin of wild-type or VDR knockout (KO) mice. *P values≤0.05

Previous studies in our laboratory have shown that VDR is a direct target of p63, however, no studies to date have examined whether VDR in turn can regulate ΔNp63*α*.^30, 31^ Since, it has been suggested that both ΔNp63*α* and VDR/VD_3_ can lead to increased keratinocyte proliferation,^8, 9, 32, 33^ we examined whether VDR was mediating cell proliferation by regulating ΔNp63*α* levels. We silenced VDR in two keratinocyte cell lines (HaCaT and HaCaT II-4) and examined whether ΔNp63*α* expression at both the protein and transcript levels were altered. To rule out p53-dependent effects, we also studied the effects of VDR silencing in primary neonatal human epidermal keratinocytes expressing wild-type p53. Cells transfected with siRNA against VDR showed a significant reduction in the transcript and protein levels of VDR (Figures 1a and b). Knockdown of VDR in HaCaT, HaCaT II-4 and neonatal human epidermal keratinocytes led to a concomitant reduction in ΔNp63*α* transcript and protein levels (Figures 1a and b). Similar results were observed in A431 cells, a SCC cell line (Supplementary Figure 1a). To further confirm that VDR is positively regulating ΔNp63*α*, we measured p63 transcript levels in total RNA obtained from skin of VDR knockout mice and wild-type littermates (obtained from Dr. Glendon Zinser at University of Cincinnati). Ablation of VDR significantly reduced the transcript levels of p63 in the skin of VDR knockout mice when compared with wild-type mice (Figure 1c). These data clearly demonstrate that VDR positively regulates ΔNp63*α* expression *in vitro* and *in vivo*.

### ΔNp63*α* protein levels increased following treatment with low dose VD_3_

**Figure 2:**
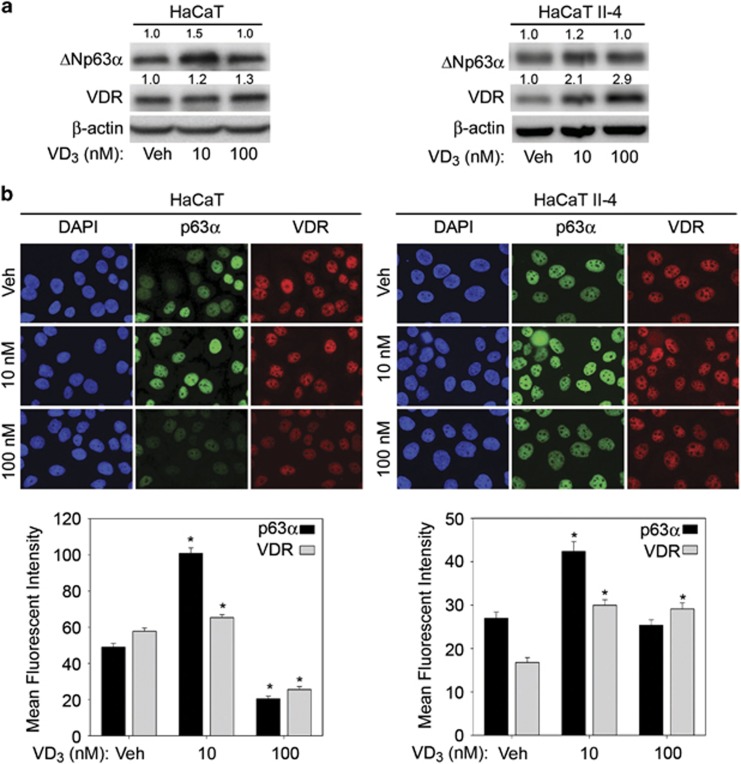
VD_3_ dosage differentially affects ΔNp63*α*. (**a**) HaCaT and HaCaT II-4 cells were treated with vehicle, 10 nM VD_3_ or 100 nM VD_3_ for 24 h, and then subjected to immunoblot analysis for ΔNp63*α*, VDR and *β*-actin. The fold change in protein levels, relative to vehicle-treated cells, is listed above each band. (**b**) Top panel: HaCaT and HaCaT II-4 were treated with vehicle, 10 nM VD_3_ or 100 nM VD_3_ overnight followed by detection of p63*α* and VDR by immunofluorescence. Bottom panel: average mean fluorescent intensity of immunofluorescence staining for p63*α* and VDR in HaCaT and HaCaT II-4. Error bars represent standard error of the mean. *P values≤0.05 compared with vehicle control cells

VDR can exert its effect in both a ligand-dependent or -independent manner.^34, 35^ Having demonstrated that VDR is essential for maintaining basal expression of ΔNp63*α*, we examined whether VDR exerts it effect on ΔNp63*α* in a ligand-dependent or -independent manner. We assessed the effects of increasing doses of VD_3_ on ΔNp63*α* expression and observed a dose-dependent increase in ΔNp63*α* levels up to 10 nM (Supplementary Figure 2a). We focused on testing the effects of 10 nM and 100 nM of VD_3_ on ΔNp63*α* expression in HaCaT, HaCaT II-4 and A431 cells for subsequent studies. Whereas treatment with low dose VD_3_ increased ΔNp63*α* protein levels in HaCaT, HaCaT II-4 and A431 cells (Figure 2a and Supplementary Figure 1b), high dose VD_3_ did not significantly affect ΔNp63*α* protein levels when compared with vehicle control treated cells (Figure 2a). Consistent with immunoblot analysis, quantitation of immunofluorescent staining of p63 and VDR in cells treated with VD_3_ clearly demonstrated an increase in ΔNp63*α* expression by 10 nM VD_3_ when compared with 100 nM VD_3_ or vehicle-treated cells (Figure 2b). These results establish that only low doses of VD_3_ leads to increased protein expression of ΔNp63*α*.

### VD_3_ increases ΔNp63*α* transcript level

**Figure 3:**
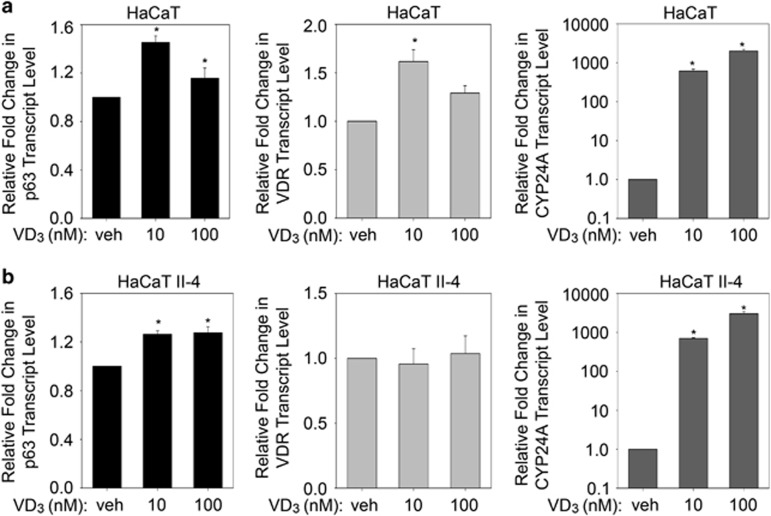
VD_3_ increases ΔNp63*α* transcript levels. (**a**) HaCaT and (**b**) HaCaT II-4 cells were treated with vehicle, 10 nM VD_3_ or 100 nM VD_3_ for 24 h. Transcript levels of p63 (left panel), VDR (middle panel) and CYP24A (right panel) were analyzed by TaqMan-based qRT-PCR (*P values≤0.05)

To understand the mechanism behind VD_3_-mediated regulation of ΔNp63*α*, we examined whether VD_3_ treatment affects ΔNp63*α* transcription. To test this, we measured p63, VDR and CYP24A transcript levels in HaCaT (Figure 3a) and HaCaT II-4 (Figure 3b) cells following treatment with 10 nM or 100 nM VD_3_ for 24 h. Both concentrations of VD_3_ led to a modest but significant increase in p63 transcript levels when compared with vehicle-treated control samples. VD_3_ did not significantly alter VDR transcript levels at 100 nM VD_3_ in HaCaT and at both doses tested in HaCaT II-4. As a positive control, we measured the transcript levels of CYP24A, a known target of VD_3_, which showed a dose-dependent increase following VD_3_ treatment. Taken together, both high and low dose of VD_3_ increased p63 transcript levels.

### Effects of VD_3_ treatment on cell proliferation correlates with ΔNp63*α* protein levels

**Figure 4:**
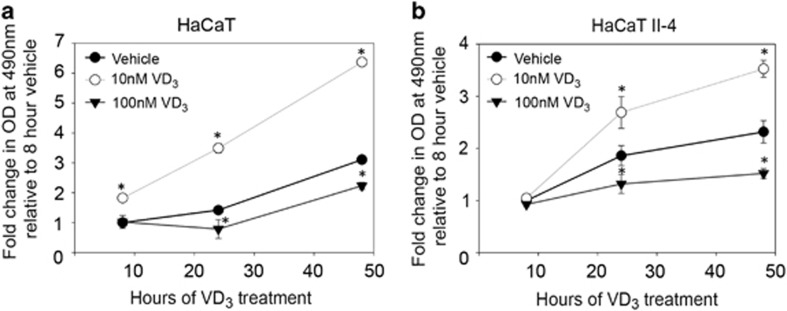
VD_3_ dosage differentially affects cell proliferation. HaCaT (**a**) and HaCaT II-4 (**b**) cells were treated with vehicle, 10 nM VD_3_ or 100 nM VD_3_ for 8, 24 and 48 h, and cell proliferation was measured by MTS cell titer assay. *Y* axis represents fold change when compared with vehicle-treated cells. Error bars represent standard deviation from the mean. *P values≤0.05 compared with vehicle control cells

Previous studies have shown a correlation between increased ΔNp63*α* expression and cell proliferation in SCCs.^26, 27, 28^ Because low dose VD_3_ increased ΔNp63*α* levels (Figures 2 and 3), we examined whether low dose VD_3_ treatment also leads to increased keratinocyte proliferation. We monitored cell proliferation at various time points following treatment of cells with VD_3_ using MTS assay and trypan blue exclusion. As seen in Figure 4, 10 nM VD_3_ significantly increased proliferation of both HaCaT and HaCaT II-4 cells, whereas 100 nM VD_3_ reduced cell proliferation when compared with vehicle-treated cells. We further confirmed dose-dependent effects of VD_3_ on increased cell proliferation by trypan blue exclusion in HaCaT and HaCaT II-4 cells treated with 1, 10 and 100 nM doses of VD_3_ (Supplementary Figures 2b and c). Consistent with MTS assay, which measures the amount of actively metabolizing cells (Figure 4), cell viability measurements by trypan blue exclusion confirmed increased cell proliferation with low dose of VD_3_, whereas 100 nM of VD_3_ led to a reduction in cell number when compared with vehicle-treated cells. Together our results demonstrate that dose-dependent effects of VD_3_ on cell proliferation correlate with the effects of VD_3_ on ΔNp63*α* levels as observed in Figure 2.

### VDR and p63 are required for VD_3_-mediated cell proliferation

**Figure 5:**
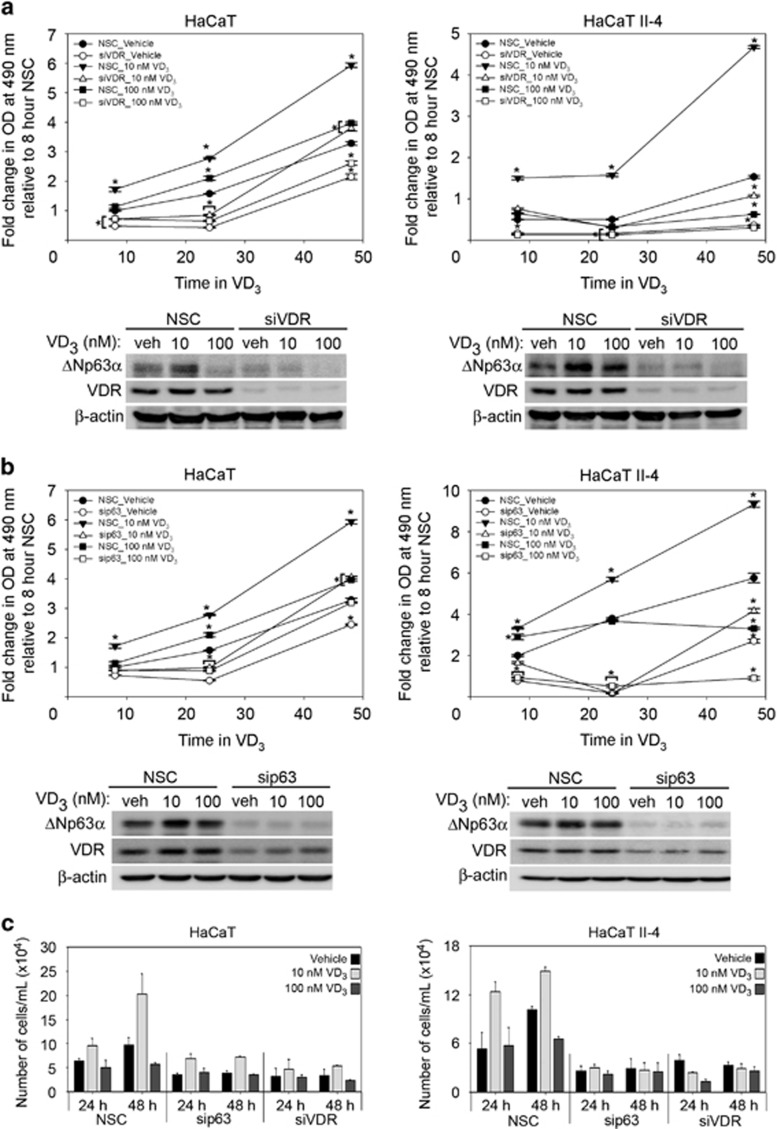
VDR and ΔNp63*α* are required for VD_3_-mediated cell proliferation. HaCaT and HaCaT II-4 cells were transfected with non-silencing control (NSC) or siVDR (panel **a**) or sip63 (panel **b**) followed by treatment with vehicle control, 10 nM or 100 nM VD_3_ for 8, 24 and 48 h as indicated. Cell proliferation was measured by MTS cell titer assay. *Y* axis represents fold change when compared with NSC transfected vehicle-treated cells. Confirmation of silencing was measured by western blot following VD_3_ treatment (lower panels). (**c**) HaCaT and HaCaT II-4 cells were transfected with siRNA against p63 or VDR followed by treatment with vehicle control or VD_3_ at 10 nM or 100 nM for 24 and 48 h. Cell viability was measured by trypan blue cell exclusion. Error bars represent standard deviation from the mean. *P values≤0.05 for knockdown condition that is significantly different from vehicle-treated NSC

VD_3_ was previously shown to function independent of its receptor, VDR.^36^ To determine whether VD_3_-mediated effects on keratinocyte proliferation are dependent on VDR, we examined the effects of VDR knockdown on cell proliferation following VD_3_ treatment. HaCaT and HaCaT II-4 cells were transfected with non-silencing control siRNA or VDR-specific siRNA followed by VD_3_ treatment. Knockdown of VDR dramatically reduced cell proliferation in both HaCaT and HaCaT II-4 cells, regardless of VD_3_ dose when compared to control siRNA-transfected cells (Figure 5a, upper panel). Immunoblot analysis of cells treated with VD_3_ following knockdown of VDR showed a reduction in basal ΔNp63*α* levels and significantly impaired the induction of ΔNp63*α* protein by treatment with 10 nM VD_3_ when compared with control cells (Figure 5a, bottom panel). Taken together, these data show that VD_3_ requires VDR to increase ΔNp63*α* and keratinocyte proliferation.

To confirm that decreased ΔNp63*α* expression observed after knockdown of VDR was responsible for the concomitant reduced proliferation, p63 was silenced prior to VD_3_ treatment of HaCaT and HaCaT II-4 cells. Silencing p63 reduced cell proliferation at all doses of VD_3_ when compared with cells transfected with control siRNA, indicating that p63 is required for VD_3_-mediated increases in cell proliferation (Figure 5b, top panel). Immunoblot analysis of cells treated with VD_3_ following knockdown of p63 demonstrated that silencing p63 also reduced VD_3_-mediated increases in VDR (Figure 5b, bottom panel). To confirm that the change in cell proliferation was not an artifact of the MTS assay, we also measured the change in cell viability following VD_3_ treatment of HaCaT and HaCaT II-4 that were transfected with siRNA against p63 or VDR by trypan blue exclusion. Consistent with MTS assay, the loss of p63 or VDR led to a reduction in VD_3_-mediated increase in proliferation by trypan blue exclusion further confirming that both p63 and VDR are required for VD_3_-mediated increase in cell proliferation (Figure 5c).

### ΔNp63*α* rescues VD_3_-mediated increase in proliferation following the loss of VDR

**Figure 6:**
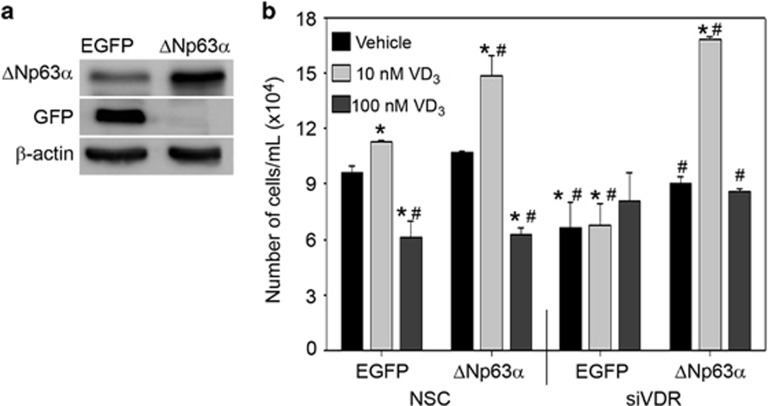
ΔNp63*α* rescues reduction in VD_3_-mediated cell proliferation following loss of VDR. (**a**) The expression of eGFP and ΔNp63*α* were confirmed in HaCaT-eGFP and HaCaT-ΔNp63*α* stable cells via immunoblot analysis. (**b**) HaCaT-eGFP and HaCaT-ΔNp63*α* stable pools were transfected with non-silencing control (NSC) or siVDR followed by treatment with vehicle control, 10 nM or 100 nM VD_3_ for 24 h. Cell viability was measured following VD_3_ treatment by trypan blue cell exclusion. Error bars represent standard deviation from the mean. *P values≤0.05 compared with vehicle control EGFP expressing HaCaT cells. ^#^*P* values≤0.05 compared with 10 nM EGFP expressing HaCaT cells

To verify that increased cell proliferation following low dose VD_3_ treatment was dependent on VDR-mediated regulation of ΔNp63*α* expression, we generated HaCaT stable cell lines expressing ΔNp63*α* (HaCaT-ΔNp63*α*) or eGFP (HaCaT-eGFP) as a control. We confirmed increased expression of ΔNp63*α* in HaCaT-ΔNp63*α* when compared with HaCaT-eGFP and only HaCaT-eGFP expressed GFP protein (Figure 6a). We next silenced VDR expression prior to VD_3_ treatment of HaCaT-eGFP and HaCaT-ΔNp63*α* cells. As shown in Figure 6b, 10 nM VD_3_ led to an increased cell number by trypan blue exclusion in both HaCaT-eGFP and HaCaT-ΔNp63*α* cells transfected with NSC when compared with vehicle treatment cells. As shown earlier (Figure 5), the loss of VDR led to a reduction in HaCaT-eGFP cell number regardless of VD_3_ dose (Figure 6b). Importantly, ΔNp63*α* rescued the effects of VDR knockdown in HaCaT-ΔNp63*α* cells treated with 10 nM VD_3_ (Figure 6b). Taken together, our results confirm that increased keratinocyte proliferation following treatment with low dose VD_3_ is dependent on VDR-mediated regulation of ΔNp63*α* expression.

### VD_3_ mediated increase in ΔNp63*α* levels occurs via p38 and Akt activation

**Figure 7:**
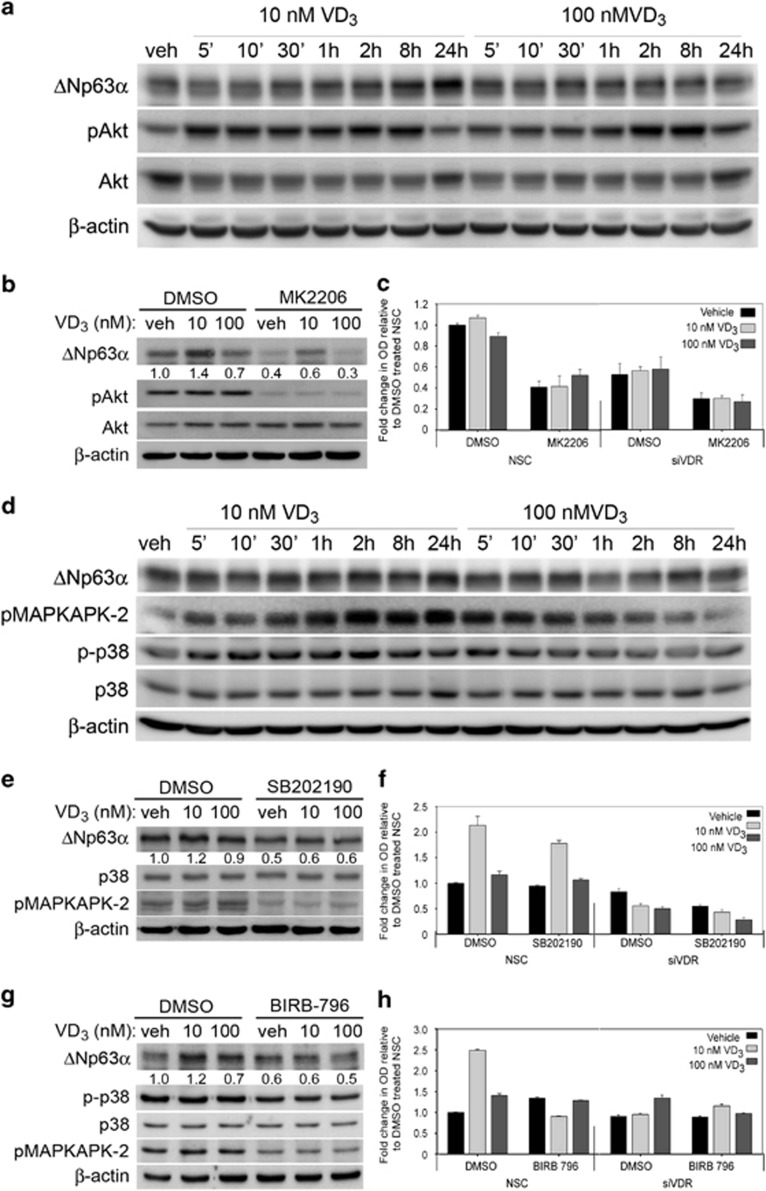
VD_3_ regulates ΔNp63*α* levels via p38 and Akt activation. (**a**) HaCaT cells were treated with VD_3_ and harvested at different time points as indicated. Whole-cell lysates were subjected to immunoblot analysis for p63, pAkt, Akt and *β*-actin. (**b**) HaCaT cells were pretreated with 10 *μ*M MK2206 or DMSO control for 1 h followed by treatment with either vehicle, 10 nM VD_3_ or 100 nM VD_3_ for 24 h. Whole-cell lysates were subjected to immunoblot analysis for the indicated proteins. The fold change in protein levels for ΔNp63*α*, relative to vehicle-treated cells, as described in materials and methods is listed below each band. (**c**) HaCaT cells were transfected with non-silencing control (NSC) or siRNA against VDR. Cells were incubated for 1 h in media containing 10 *μ*M MK2206 or DMSO control prior to treatment with the indicated doses of VD_3_ or vehicle for 24 h and MK2206 or DMSO. Cell proliferation was measured 24 h post VD_3_ treatment by MTS assay. (**d**) HaCaT cells were treated with VD_3_ and harvested at different time points as indicated. Whole-cell lysates were subjected to immunoblot analysis for p-p38, p63, pMAPKAPK-2, p38 and *β*-actin. (**e** and **g**) HaCaT cells were pretreated with 15 *μ*M SB202190 or DMSO control for 1 h (**e**) or pretreated with 1 *μ*M BIRB-796 or DMSO control for 2 h (**g**) followed by treatment with either vehicle, 10 nM VD_3_ or 100 nM VD_3_ for 24 h. Whole-cell lysates were subjected to immunoblot analysis for the indicated proteins. The fold change in protein levels for ΔNp63*α*, relative to vehicle-treated cells, are listed below each band. (**f** and **h**) HaCaT cells were transfected with NSC or siRNA against VDR. Cells were pretreated with 15 *μ*M SB202190 or DMSO control for 1 h (**f**) or pretreated with 1 *μ*M BIRB-796 or DMSO control for 2 h (**h**) prior to replacing media with fresh SB202190 or BIRB-796 and either vehicle, 10 nM VD_3_ or 100 nM VD_3_ for 24 h. Cell proliferation was measured 24 h post VD_3_ treatment by MTS assay

Previous studies have shown increased Akt activation following VD_3_ treatment, particularly low doses of VD_3_.^8, 9^ The Akt pathway has been shown to increase ΔNp63*α* protein expression.^13^ We therefore wanted to examine whether Akt activation upon VD_3_ treatment was required for increased ΔNp63*α* protein levels. We measured Akt activation after treatment with VD_3_ using an antibody directed against phosphorylated serine 473 of Akt (pAkt) and observed activation of Akt by low dose VD_3_ preceded the increase in ΔNp63*α* (Figure 7a). To confirm that increased ΔNp63*α* levels and cell growth observed upon low dose VD_3_ treatment is occurring because of activated Akt, we next examined the effects of VD_3_ on ΔNp63*α* levels following the inhibition of Akt activity with small molecule Akt specific inhibitor, MK2206. Inhibition of Akt activity reduced ΔNp63*α* levels in all conditions (Figure 7b). However, low dose VD_3_ treatment in conjunction with MK2206 still exhibited slightly higher levels of ΔNp63*α* when compared with cells treated with MK2206 and vehicle (Figure 7b). We studied the effects of MK2206 pre-treatment on cell proliferation in presence or absence of VDR in HaCaT cells. Loss of VDR alone or inhibition of Akt alone reduced HaCaT proliferation to the similar levels regardless of VD_3_ dose (Figure 7c). Inhibition of Akt combined with loss of VDR led to a further reduction in cell proliferation. Taken together, these results suggest that it is likely that in addition to Akt activation by VD_3_, increased ΔNp63*α* protein levels following low dose VD_3_ treatment occurs via yet another pathway.

We next examined the role of p38 mitogen-activated protein kinase (MAPK) signaling pathway in VD_3_-mediated increase in ΔNp63*α* levels. In addition to VD_3_ treatment, a variety of other stimuli such as stress, growth factors or cytokines leads to the activation of p38 MAPK.^10, 11^ Interestingly, activated p38 MAPK was shown to increase ΔNp63*α* levels and cell proliferation in limbal epithelial cells.^12^ To assess p38 MAPK activation by VD_3_ in HaCaT cells following treatment with 10 nM and 100 nM of VD_3_, we monitored the phosphorylation of p38 MAPK and its downstream target MAPKAPK-2 at various time points post treatment. VD_3_ induced the phosphorylation of both p38 MAPK and MAPKAPK-2 within 5 min of VD_3_ treatment, which lasted longer following treatment with 10 nM VD_3_ when compared with 100 nM VD_3_ treatment (Figure 7d). Consistent with Figure 6a, we observed an increase in ΔNp63*α* protein levels following kinase activation with 10 nM VD_3_ (Figure 7d). To confirm that VD_3_ increased ΔNp63*α* protein levels via p38 MAPK activation, we examined whether pre-treatment with the p38 MAPK inhibitors SB202190 or BIRB-796 prior to VD_3_ treatment led to a decrease in ΔNp63*α* protein levels. Inhibition of p38 activity led to a reduction in the ΔNp63*α* protein levels (Figures 7e and g). Furthermore, low dose VD_3_ was unable to rescue ΔNp63*α* levels when p38 MAPK activation was inhibited (Figures 7e and g), indicating that increased ΔNp63*α* levels in response to low levels of VD_3_ occur via p38 MAPK activation.

Next, we studied the effects of SB202190 or BIRB-796 pre-treatment on cell proliferation in the presence or absence of VDR in HaCaT cells. Inhibition of p38 MAPK led to a reduction in cell proliferation of cells treated with 10 nM VD_3_ compared with cells treated with high dose VD_3_ and DMSO (Figures 7f and h). The loss of VDR alone or in combination with p38 MAPK inhibition reduced keratinocyte proliferation to similar levels regardless of VD_3_ treatment (Figures 7f and h). Taken together, these data clearly indicated that p38 MAPK is required for low dose VD_3_-mediated increase in ΔNp63*α* protein levels and cell proliferation.

### VDR and p63 expression are increased in NMSC

**Figure 8:**
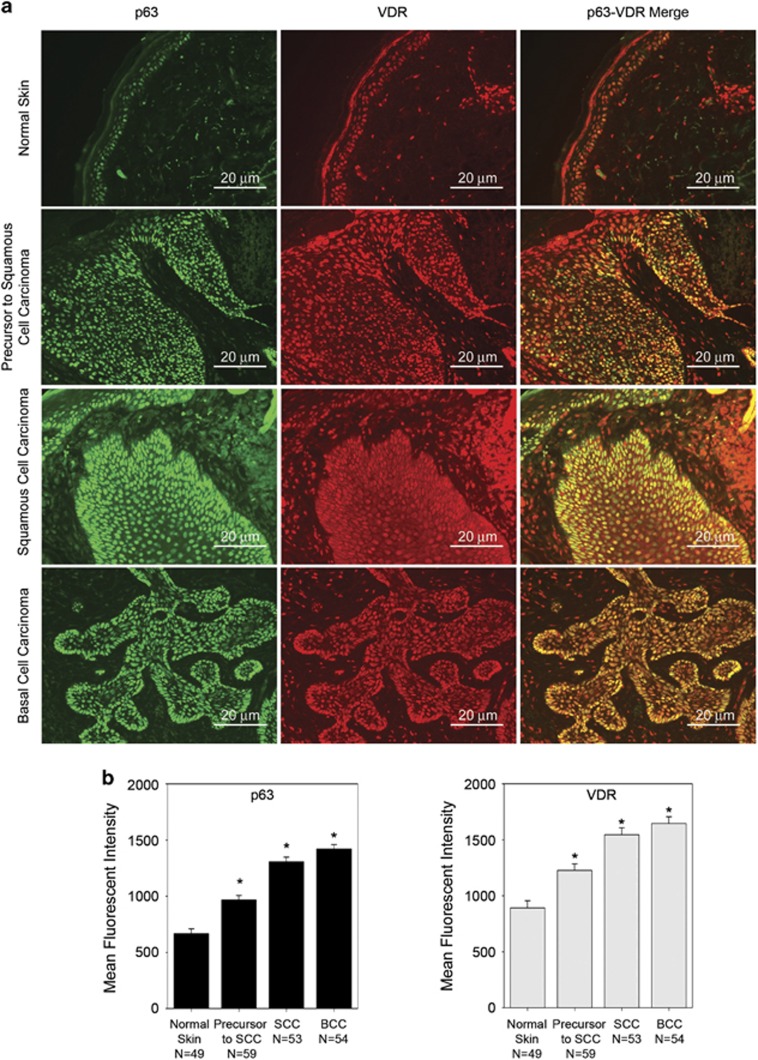
VDR and p63 expression are increased in NMSC. (**a**) Top panels show representative images taken at a × 20 magnification of normal skin, precursors to SCC, SCC and BCC from formalin fixed, paraffin-embedded human skin stained for p63 and VDR (scale bar=20 *μ*m). (**b**) Quantitation of p63 and VDR levels from 49 normal skin samples, 59 precursors to SCC samples, 53 SCC samples and 54 BCC samples are plotted. *Y* axis represents the mean fluorescent intensity, normalized to background, in arbitrary units. Error bars represent standard error. *P≤0.05 compared with normal skin

ΔNp63*α* is required for increased keratinocyte proliferation and has been shown to have a role in maintaining the proliferative capacity of basal keratinocytes *in vivo.*^15, 18, 19, 20, 21, 22^ Both VDR and p63 have been shown to be overexpressed in NMSCs.^23, 24, 25, 37, 38, 39, 40^ Therefore, we examined the expression levels of p63 and VDR in paraffin-embedded skin samples from individuals with normal skin (*N*=49) and patients with BCCs (*N*=54), SCC (*N*=53) and the precursors to SCC (*N*=59) by immunofluorescence to determine whether there is a correlation between p63 and VDR expression levels. We observed significantly higher levels of both p63 and VDR in BCC, SCC and the precursors to SCC when compared with normal non-cancerous skin as shown in representative images (Figure 8a). The levels of both p63 and VDR were highest in BCC followed by SCC and precursors to SCC (Figure 8b). Increased level of p63 and VDR might be responsible for increased proliferation upon exposure to VD_3_ in NMSC. Interestingly, VD_3_ production in the skin is a result of UV exposure and UV radiation is the most common cause of these cancers.

## Discussion

An initial stage of proliferation followed by concomitant differentiation and inhibition of proliferation are important features of the skin, allowing for the development of each layer of the skin.^41^ Delineating the mechanisms involved in triggering or preventing keratinocyte proliferation remains critical for a better understanding of NMSC. ΔNp63*α* is the most abundantly expressed p63 isoform in the basal layer of the skin where it is well known to upregulate genes involved in proliferation.^14, 16^ We previously showed that ΔNp63*α* positively regulates VDR expression.^30^ VDR is also expressed in the basal layer of the skin and is well known for its role in differentiation and calcium homeostasis.^35, 42^ Previous work showed that VDR functioned independently of VD_3_ to activate basal transcription of 24-hydroxylase.^43^ In support of the ligand-independent effects of VDR, we have shown that VDR is essential for basal levels of ΔNp63*α* transcription (Figure 1) and could also increase the expression of ΔNp63*α* in the presence of its ligand VD_3_ (Figure 2). Our findings suggest a new role for VDR in proliferation and cell survival through the regulation of ΔNp63*α*. Here, we show that VD_3_ can regulate ΔNp63*α* and increase keratinocyte proliferation (Figures 2 and 4). The ligand requirement of VDR-mediated transcription might be context-specific allowing VDR to regulate genes necessary for cell homeostasis in the presence or absence of VD_3_, providing an additional layer by which keratinocyte proliferation is maintained in response to varying environmental conditions.

VDR and VD_3_ regulate many transcriptional targets, but their regulation of cellular processes is not limited to transcription; VDR and VD_3_ also have non-genomic processes in the cell.^34^ Several non-genomic functions of VD_3_ have previously been determined, some of these functions included insulin secretion, smooth muscle migration and opening calcium and chloride channels.^34^ We demonstrated that VD_3_ enhances ΔNp63*α* expression in a non-genomic manner by increasing ΔNp63*α* protein within hours of VD_3_ treatment (Figures 7a and d). This increase in ΔNp63*α* protein levels was shown to be dependent on VD_3_ concentration as only low dose VD_3_ was able to increase ΔNp63*α* levels (Figure 2 and Supplementary Figures 1 and 2). Furthermore, this increase in ΔNp63*α* protein levels by low dose VD_3_ correlated to an increase in cellular proliferation (Figure 4), whereas knockdown of ΔNp63*α* or VDR reduced cell proliferation at all doses tested (Figure 5). Overexpression of ΔNp63*α* is seen in many NMSCs, such as SCC and VD_3_ has been considered a chemotherapeutic adjuvant.^4, 6, 7, 12, 23, 26, 27^ Our study indicates that reduction in ΔNp63*α* levels could improve the therapeutic potential of VD_3_ in treating NMSCs. This is supported by previous studies, which have also established that ΔNp63*α* mediates resistance to cisplatin in head and neck SCCs and breast cancers, demonstrating the widespread benefit to controlling ΔNp63*α* in the treatment of epithelial cancers.^44, 45, 46^

Prior studies have shown that VD_3_ can activate p38 MAPK via non-genomic signaling by binding VDR complexed with caveolin-1 at the plasma membrane.^10, 11^ Once VD_3_ is bound to membrane-associated VDR and caveolin-1, Src is recruited for the activation of p38 MAPK.^10^ Activated p38 MAPK has been shown to increase ΔNp63*α* protein levels.^11^ Our study shows that inhibition of p38 MAPK activation attenuates low dose VD_3_-mediated increases in ΔNp63*α* levels (Figure 7e and g). COX-2 a target of p38 MAPK is upregulated in NMSC, which further supports our findings that p38 MAPK is active in these cancers and can regulate its target genes, such as ΔNp63*α*.^47^ Our data indicate that inhibition of p38 MAPK leads to a decrease in VD_3_-mediated increases in ΔNp63*α*, a marker of non-melanoma skin, suggesting that p38 MAPK is a potential pathway to target, in conjunction with chemotherapeutic regiment in these cancers.

We demonstrated that both p63 and VDR expression are increased in BCC, SCC and the precursor lesions to SCC (Figure 8). Given the role of p63 and VDR in the inhibition of cell invasion, it came as a surprise that both VDR and p63 show high expression in the more invasive cancer, SCC, when compared with the precursor of SCC (Figure 8). Consistent with previous reports, p63 show increased expression in BCC,^18^ which correlates with an increased expression of VDR in BCC as shown in Figure 8. Our results imply that an elevated level of VDR does not indicate a good prognosis because we observed higher levels of VDR in more invasive cancers. p63 and VDR expression could be used as a predictor of patient response to VD_3_ treatment as a chemotherapeutic agent. We have shown that VDR and VD_3_, known for their role in cancer prevention, also have a role in the regulation of oncogenic ΔNp63*α*, thus increasing keratinocyte proliferation at low VD_3_ concentration, bringing to light a drawback of the use of VD_3_ in cancer prevention and treatment.^48, 49^ Altogether these results suggest that VD_3_ can have either a growth-suppressive or growth-stimulatory role in the presence of VDR in keratinocytes cells by regulating the expression of ΔNp63*α*. Monitoring VD_3_ concentration in cancer treatment regimens will be critical to induce the proper signaling pathway to reduce cancer cell growth especially because our recent study clearly demonstrated that tumors from SKH-1 mice showed an increase in p63 expression with increasing concentration of dietary vitamin D.^50^ Since, VD_3_ action is dose-dependent, ensuring high doses of VD_3_ are getting into cancer cells to reduce proliferation and ΔNp63*α* expression will be critical to its use as a chemotherapeutic agent.

## Materials and Methods

### Cell lines and reagents

The non-tumorigenic immortalized human keratinocyte HaCaT cell line was obtained from Dr. Nancy Bigley (Wright State University), while the tumorigenic H-Ras transformed HaCaT II-4 cells were obtained from by Dr. Nancy Colburn (National Cancer Institute). The SCC cell line A431 was obtained from ATCC (Manassas, VA, USA). The three cell lines were maintained in Dulbecco's modified Eagle's medium supplemented with 8% fetal bovine serum and 250 U penicillin and 250 *μ*g streptomycin. Neonatal human epidermal keratinocytes were maintained in KGM-Gold media as per manufacturer's instructions (Lonza, Walkersville, MD, USA). The Akt inhibitor MK2206 and the p38 inhibitor BIRB-796 were purchased from Selleckchem (Houston, TX, USA). The p38 inhibitor SB202190 was purchased from Cell Signaling Technology (Danvers, MA, USA). VD_3_ was maintained as a 100 *μ*M a stock in 100% ethanol (Cat # 17936, Sigma-Aldrich, St. Louis, MO, USA). VD_3_ treatment were carried out in Dulbecco's modified Eagle's medium supplemented with 8% Charcoal-striped fetal bovine serum and 250 U penicillin and 250 *μ*g streptomycin with a final EtOH concentration of 0.01%.

### Knockdown and overexpression

VDR and p63 knockdown studies conducted in HaCaT, HaCaT II-4, A431 and neonatal human epidermal keratinocyte cells were performed by two rounds of siRNA transfection using Lipofectamine RNAi-Max as per the manufacturer's instructions (Invitrogen, Carlsband, CA, USA). VDR and p63 siRNA used in this study were purchased from Qiagen (Valencia, CA, USA) and the target sequences used were described earlier.^30^ The HaCaT-eGFP and HaCaT-ΔNp63*α* stable cell lines were generated by infecting parental HaCaT cells with lenti-virus plasmids expressing eGFP or ΔNp63*α* as described earlier.^30^ At 72 h post infection, transduced cells were selected in blasticidin (10 mg/ml) to obtain HaCaT stable cells expressing eGFP or ΔNp63*α*.

### Reverse transcription PCR

Total RNA was extracted from human cells using the eZNA RNA isolation kit according to the manufacturer's protocol (Omega Bio-Tek, Norcross, GA, USA). TaqMan reverse transcription kit (Applied Biosystems, Foster City, CA, USA) was used to synthesize cDNA from 1*μ*g of total RNA. Total RNA extracted from the skin of VDR knockout mice and wild-type littermates were provided by Dr. Glendon Zinser (University of Cincinnati) in full accordance with the Institutional Animal Care and Use Committee of the University of Cincinnati. Quantitative real-time PCR analysis was performed as previously described using Assay on Demand specific for the genes of interest and normalized to endogenous GAPDH for human genes or to *β*-actin for murine genes of interest (PE Applied Biosystems, Foster City, CA, USA).^30, 51^ Human Assays on Demand used were GAPDH (4325792), VDR (Hs_0017213_ml) and pan p63 (Hs_00978340_ml). Murine Assays on Demand were pan p63 (Mm00495788_m1), VDR (Mm00437297_m1) and β-actin (Mm00607939_s1). Each experiment had an *n*=3 independent experiments. Student's *t*-tests were used to determine significant difference.

### Cell immunofluorescence assay

Cells were grown on sterile glass coverslips prior to fixation with 2% paraformaldehyde for 15 min. After three consecutive washes with PBS, cells were permeabilized with 0.2% triton X-100 diluted in PBS for 5 min. Cells were washed and blocked with 0.5% normal goat serum in PBS thrice before incubating with rat monoclonal anti-VDR 9A7 (Sigma-Aldrich) and rabbit polyclonal anti-p63 H129 (Santa Cruz Biotechnology, Santa Cruz, CA, USA) antibodies primary antibodies for 1 h at room temperature. Excess primary antibody was removed with three consecutive 5-min washes in PBS-normal goat serum followed by incubation with AlexaFluor goat anti-rabbit 488 and goat anti-rat 568 antibodies at a dilution of 1 : 500 for 1 h at room temperature. Excess secondary antibody was removed with three consecutive 5-min washes in PBS-normal goat serum and one wash in PBS prior to mounting with Vecta-Shield plus DAPI Mounting Media (Vector Laboratories, Burlingame, CA, USA). Cells were visualized and imaged using a Leica CTR 6000 Microscope (Leica Microsystems, Wetzlar, Germany) and ImagePro 6.2 software (Media Cybernetics, Bethesda, MD, USA). Mean fluorescent intensity was determined with the ImagePro 6.2 software after normalizing to background fluorescence. At least 100 cells were measured for VDR and p63 staining intensity per condition for each experiment with an *n*=3 independent experiments. Student's *t*-tests were used to determine significant difference.

### Immunoblot analysis

Whole-cell lysates were prepared by lysing the cells in phosphatase inhibitors containing buffer (50 mM Tris-HCl pH 8, 120 mM NaCl, 5 mM NaPPi, 10 mM NaF, 30 mM paranitrophenylphosphate, 1 mM benzamidine, 0.1% NP-40, 1% Triton X-100 and 0.2 PMSF, 100 nM sodium orthovanadate) supplemented with protease inhibitor cocktail (Sigma, St. Louis, MO, USA). Total protein concentrations were determined by BCA protein detection method (Thermo Fisher Scientific Inc., Fremont, CA, USA). Equivalent concentration of proteins were resolved on 10% SDS-PAGE and transferred to polyvinylidene difluoride membranes. Proteins were detected using the following antibodies: rabbit polyclonal anti-phospho-Akt (Ser473), rabbit polyclonal anti-Akt, rabbit polyclonal anti-p38, rabbit polyclonal anti-phospho-p38 (Thr180/Tyr182), rabbit monoclonal anti-phospho-MAPKAPK-2 (Thr222) (Cell Signaling Technology), mouse monoclonal anti-VDR D-6, mouse monoclonal anti-pan p63 4A4 and mouse monoclonal anti β-actin (Santa Cruz Biotechnology) and rabbit polyclonal anti-GFP (FL) (Santa Cruz Biotechnology) obtained from the laboratory of Dr. Michael Leffak at Wright State University. Appropriate horseradish peroxidase-conjugated secondary antibodies (Promega, Madison, WI, USA) were used for chemiluminescence detection with Western Lightning Plus chemiluminescent kit (Perkin Elmer, Waltham, MA, USA). Fold change in protein expression was calculated by normalizing band intensity to β-actin followed by determination of the intensity change from vehicle or NSC.

### Cell growth assays

Cells were seeded at 5000 cells per well in a 96-well flat bottom culture dish and at 24 h post-plating, cells were treated with 1 nM, 10 nM or 100 nM VD_3_ as indicated. Proliferation was measured using Promega CellTiter 96 AQ_ueous_ One Solution Cell Proliferation Assay (MTS) at various time points post-treatment as described earlier.^20^ Student's *t*-tests were used to determine significant difference. Cell viability was also measured by trypan blue exclusion and carried out in triplicate per condition per day post VD_3_ treatment.

### Tissue immunofluorescence assay

Formalin-fixed, paraffin-embedded human skin sections were stained for p63, and VDR as previously described^20, 30^ with one modification. Briefly, the human tissue were co-stained for p63 and VDR with two different antigen-retrieval processes, a heat base antigen-retrieval method was used for p63 followed by^20, 30^ an acid antigen-retrieval processes for VDR and finally neutralization with 0.1 M Borate Buffer pH8.5.^50^ Human tissue samples consisted of normal skin (*N*=49), precursor to SCC (*N*=59) which included actinic keratosis, acantholytic actinic keratosis, acantholytic and hyperplastic actinic keratosis, hyperplastic actinic keratosis, squam *in situ* and bowenoid actinic keratosis, SCC (*N*=53) which included superficial SCC, SCC arising from actinic keratosis background, SCC with perineural invasion, SCC, BCC (*N*=54) which included infiltrative BCC, nodular BCC, nodular and infiltrative BCC and superficial BCC. Tissues were imaged using a Leica CTR 6000 Microscope (Leica Microsystems). Multiple measurements (at least nine), all of the same size, were taken of the epidermal tissue or cancerous tissues for each sample. Average mean fluorescence intensity was calculated following normalization to background by using the ImagePro 6.2 software.

### Statistical analysis for stained tissue

Adjusted mean MFI and standard error of mean levels of p63 and VDR from normal skin samples, BCC samples, SCC samples and precursor to SCC samples were plotted. Repeated measures analysis of variance tests were conducted to account for the correlation between repeated measurements (nine measurements per sample). The comparisons of several covariance structures of repeated measures were performed to find the best covariance structure. The best covariance structure for the model was selected based on the smallest Akaike Information Criterion.^52^ *Post hoc* multiple comparison procedures using Dunnett's test were performed to compare between mean MFI values of p63 and VDR between control samples (i.e., normal skin samples) and all other carcinoma samples.^53, 54^ PROC MIXED procedure (SAS/STAT, Ver 9.3, SAS Institute Inc., Cary, NC, USA) was used for analyses. Maximum experiment-wise error rates of 0.05 were set to consider whether differences were statistically significant.

## Abbreviations

VDR: vitamin D receptor
VD_3_: 1*α*, 25-dihyroxyvitamin D_3_
NMSC: non-melanoma skin cancer
BCC: basal cell carcinoma
SCC: squamous cell carcinoma
MAPK: mitogen-activated protein kinase
