# Supplementary figures and images for: 1α, 25-Dihydroxyvitamin D3 and the vitamin D receptor regulates ΔNp63α levels and keratinocyte proliferation
Source: Cell Death Dis. 2015 Jun 11;6(6):e1781–. doi: 10.1038/cddis.2015.148 (PMC4669830; doi:10.1038/cddis.2015.148)

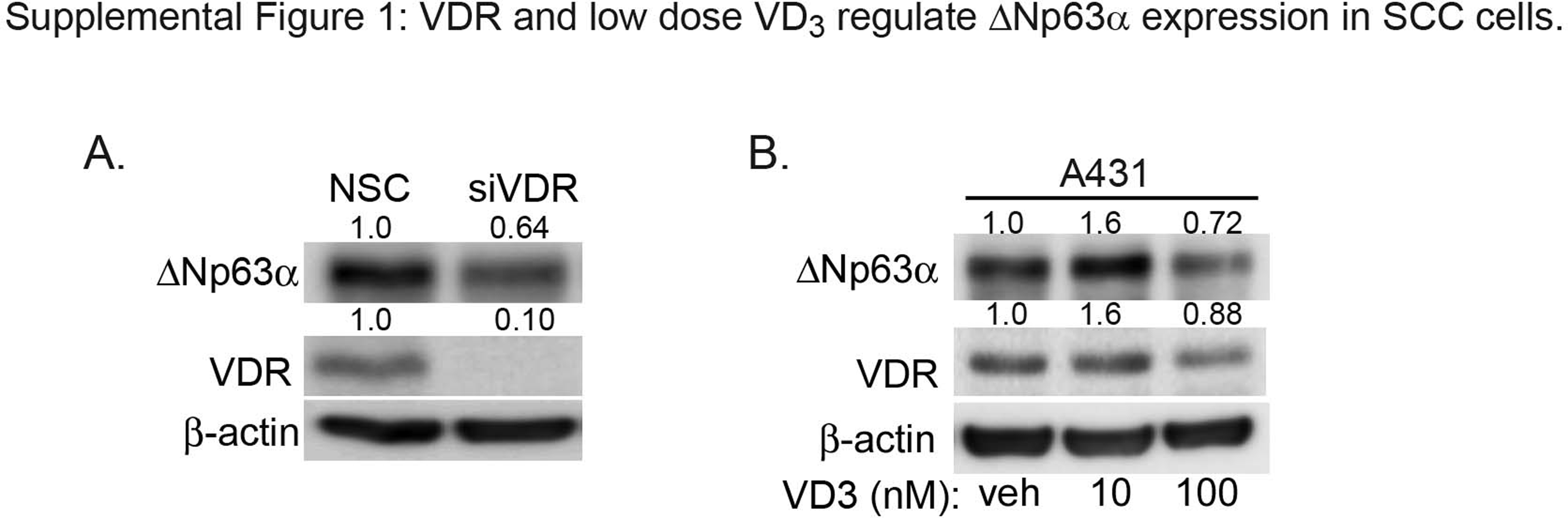

Supplement: Supplementary Figure 1 [file cddis2015148x1.tif]

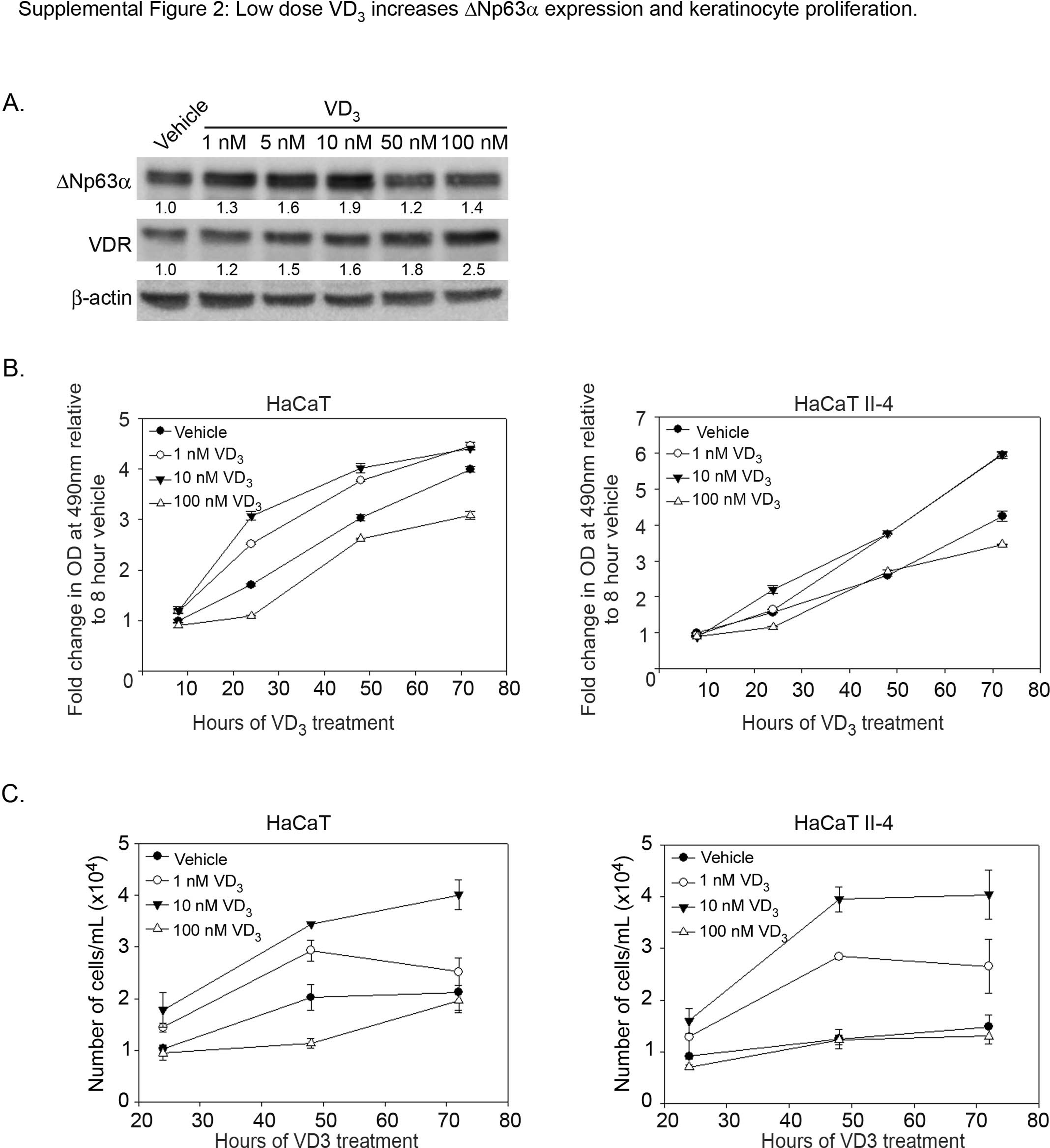

Supplement: Supplementary Figure 2 [file cddis2015148x2.tif]
